# Bilateral renal vein thrombosis in a preterm with Netherton syndrome

**DOI:** 10.1007/s00467-025-06821-2

**Published:** 2025-06-05

**Authors:** Ana Roche-Gomez, Diana Voskanyan, Joanna Śladowska-Kozłowska, Giulia Bassanese, Claus Peter Schmitt

**Affiliations:** 1 Pediatric Nephrology Department, Hospital Universitario 12 de Octubre, Madrid, Spain; 2 Pediatric Nephrology Department, Arabkir Joint Medical Center and Institute of Child and Adolescent Health, Yerevan, Armenia; 3 University of Heidelberg, Medical Faculty Heidelberg, Center for Pediatric and Adolescent Medicine, Clinic 1, Pediatric Nephrology, Heidelberg, Germany

**Keywords:** Netherton syndrome, Kidney vein thrombosis, Hypernatremia, Acute kidney injury

## Abstract

Netherton syndrome (NS) is a rare autosomal recessive syndrome caused by mutations in the serine protease inhibitor of the *Kazal type 5* (*SPINK5*) gene. Patients are characterized by the classic triad of congenital ichthyosiform erythroderma, a hair shaft abnormality termed trichorrhexis invaginata and atopic diathesis. These children require meticulous fluid and salt control from the first day of life to prevent kidney damage due to the defective skin barrier.

## Introduction

Netherton syndrome (NS) is a rare autosomal recessive disorder of cornification, resulting from mutations in the *SPINK5* gene, encoding a serine protease inhibitor on epithelial and mucosal surfaces [1–3]. Multiomics studies demonstrated upregulation of the IL-17 and IL-36 pathways. The classic triad includes congenital ichthyosiform erythroderma, trichorrhexis invaginata (“bamboo hair”) and atopic diathesis [1–4]. Clinical manifestations start during the first postpartum weeks [1–3]. NS should be considered in all infants presenting with ichthyosiform erythroderma, as 20% will have NS [1]. Frequent complications include hypernatremic dehydration, altered thermoregulation, failure to thrive, infections and sepsis [1–4]. Kidney damage may develop with cutaneous fluid and salt losses. We report a preterm boy who presented in the first days of life with acute kidney injury (AKI) due to bilateral renal vein thrombosis and subsequent chronic kidney disease (CKD).

## Case report

A 25-week preterm male infant was born with 890 g birth weight after uncomplicated pregnancy. Premature rupture of the membranes and a pathological cardiotocogram led to labor and intubation. Due to hyperbilirubinemia, phototherapy was started on day two of life. On the third day, macrohematuria, microalbuminuria and beta-2-microglobulinuria, subsequent anuria, and a serum creatinine of 3.6 mg/dl and urea of 227 mg/dl were noted. On ultrasound, both kidneys appeared hyperechogenic with abolished medulla-cortex differentiation and reduced perfusion together with a 9-mm thrombus in the right kidney vein and the inferior cava, while the left kidney vein could not be assessed. AKI stage 3 was diagnosed and the child was transferred to our center.

Upon arrival, generalized erythema and scaling were noted, suggesting congenital ichthyosis. Due to anuria and exceedingly high uremic retention parameters (serum creatinine 4.7 mg/dl, urea 219 mg/dl, phosphate 2.8 mmol/l, potassium 6.2 mmol/l), a pigtail catheter was placed for immediate peritoneal dialysis (PD), and therapy with low molecular heparin was initiated. Volume management included close monitoring of clinical status, vital signs, central venous pressure and considering cutaneous losses, ultrafiltration with PD. The child could be extubated 24 h after PD onset. A skin biopsy suggested NS, and genetic studies reconfirmed the diagnosis, identifying a homozygous *SPINK5* variant encoding the serine protease inhibitor LEKTI.

In the following days, urine excretion resumed, kidney function recovered and diuretic therapy was tapered from day 32 of life on. Intermittent sodium bicarbonate and potassium supplementation were required until day 37 of life, when PD could be discontinued. Ultrasound demonstrated improved bilateral kidney perfusion. Thrombophilia screening did not identify a specific prothrombotic risk factor. Kidney function progressively improved In the following weeks to a serum creatinine plateau from 1.47 to − 1.81 mg/dL. Serum albumin was 32–38 g/l, and electrolytes were within normal range. Urinalysis demonstrated microhematuria and low range proteinuria.

Skin symptoms improved with multiple ointments and re-lipidating cremes with fatty acids together with Vaseline and zinc oxide. Subsequent severe hyperlipidemia was related to the treatment with the re-lipidating fatty acid-based cremes, assuming major absorption across the non-functioning skin barrier. These cremes were discontinued and serum cholesterol normalized.

At 3 months of age, the boy was readmitted with oliguria caused by dehydration due to cutaneous fluid losses and preexisting residual kidney damage. Serum creatinine was 2.7 mg/dl, urea 176 mg/dl, and serum sodium 181 mmol/l. Hemofiltration was performed during 48 h for slow reversal of the severe imbalances. Fluid replacement comprised saline solution and oral rehydration via nasogastric tube. Kidney function recovered again, and 1 month later serum creatinine was 0.81 mg/dl. Subsequent episodes of dehydration were mild, and hypernatremia did not exceed 155 mmol/l and could be handled with fluid substitution.

**Fig. 1:**
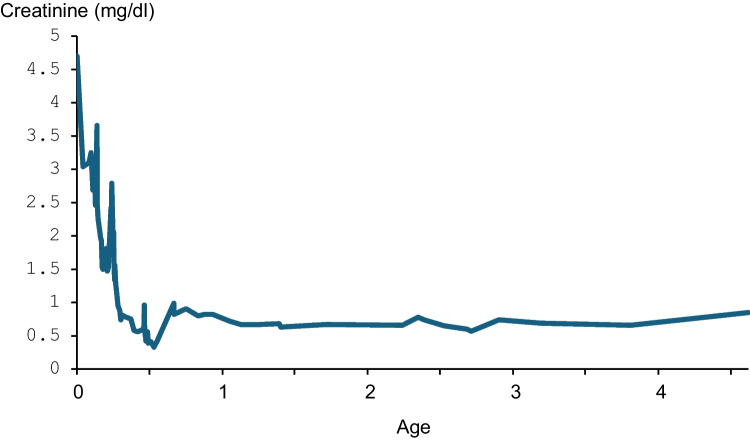
Serum creatinine in a boy with oliguric AKI at the age of three days due to bilateral kidney vein thrombosis and subsequent kidney recovery, followed by milder episodes of dehydration and AKI during the first year of life

Due to failure to thrive, a gastrostomy was placed at 5 months of age, progressively improving weight gain, but not growth rate. Therefore, growth hormone (GH) therapy was started at an eGFR of 42 ml/min/1.73 m^2^ at 2 years of age. At 5 years, the boy has a decreased but stable kidney function with a serum creatinine of 0.85 mg/dl (Fig. 1), an eGFR of 40 ml/min/1.73 m^2^, normal blood pressure, and low range proteinuria.

## Discussion

We provide a case of a preterm with NS, who presented in the first days of life with severe AKI and bilateral kidney vein thrombosis. A systematic literature review using “Netherton syndrome” and “kidney/thrombosis” identified only one additional case of bilateral kidney vein thrombosis [5]. This case developed at the age of 1 year due to repeated vomiting and fluid refusal. The present case highlights the need for adequate fluid control from the first day of life, when the diagnosis of NS can only be suspected. Prevention of bilateral kidney vein thrombosis in children with NS, however, requires utmost care for adequate high-volume fluid resuscitation from the first day of life onward, especially in preterm newborns [1–3, 5].

Our patient had several risk factors for thrombosis: dehydration due to the skin defect, aggravated by fluid losses due to phototherapy, and prematurity with low urine concentration capacity. These led to the bilateral kidney vein thrombosis. Thrombophilia screening was unremarkable. The severe degree of uremia on day three of life may be explained by two distinct pathomechanisms—bilateral kidney vein thrombosis and major dehydration—mandating PD. The thrombosis was treated with low molecular heparin, but not thrombolytic therapy in view of the bleeding risks in the preterm newborn, which is in line with expert opinions.

The second episode of severe hypernatremia and AKI, despite intense skin treatment and close follow-up, requiring hemofiltration, highlights the challenges of fluid and electrolyte balance control due to the defective cutaneous barrier even beyond the first weeks of life. Hypernatremia is the most prevalent electrolyte disturbance reported in these patients [3, 5]. Other nephrological manifestations include aminoaciduria, hypoalbuminemia, and the development of CKD mainly due to episodes of acute kidney injury associated with dehydration [3, 5].

Due to the major fluid and electrolyte losses, timely gastrointestinal tube feeding is recommended in NS, and in cases of CKD, which further impairs physical development, GH therapy may be considered [1–3]. In our case, this improved but did not normalize body weight and length. Novel therapeutic approaches, however, targeting interleukins and TNF-alpha may further improve cutaneous manifestations, growth rate, and systemic inflammation and are well tolerated [4]. Such therapy has not been initiated in our preterm patient during infancy, since at the time experience was lacking and dosing uncertain, but should be considered in future cases.

## Conclusions

Altogether, our case report and literature review illustrate the need for meticulous fluid and electrolyte control in children with NS from the first day of life onward. Otherwise, severe dehydration, hypernatremia, and secondary organ damage involving the kidney may repeatedly develop.

## Summary

### What is new?

- We provide a case report on bilateral renal vein thrombosis and a literature review on rare kidney complications in patients with NS, which illustrate the need for meticulous fluid and electrolyte control to respective sequelae.

## Data Availability

All relevant data are included in the manuscript. Additional data may be provided upon request.
